# Tumor Necrosis Factor α-Converting Enzyme Inhibition Reverses Hepatic Steatosis and Improves Insulin Sensitivity Markers and Surgical Outcome in Mice

**DOI:** 10.1371/journal.pone.0025587

**Published:** 2011-09-27

**Authors:** Vincent E. de Meijer, Hau D. Le, Jonathan A. Meisel, Anisha K. Sharma, Yury Popov, Mark Puder

**Affiliations:** 1 Department of Surgery and The Vascular Biology Program, Children's Hospital Boston, Harvard Medical School, Boston, Massachusetts, United States of America; 2 Department of Surgery, Erasmus Medical Center – University Medical Center Rotterdam, Rotterdam, the Netherlands; 3 Department of Surgery, Beth Israel Deaconess Medical Center, Harvard Medical School, Boston, Massachusetts, United States of America; 4 Division of Gastroenterology and Hepatology, Beth Israel Deaconess Medical Center, Harvard Medical School, Boston, Massachusetts, United States of America; University of Tor Vergata, Italy

## Abstract

**Background:**

Hepatic steatosis is an established risk factor for complications following major hepatic resection. Pharmacological options to reverse steatosis prior to surgery, however, are lacking. We hypothesized that treatment with the pharmacologic tumor necrosis factor-α converting enzyme (TACE)-inhibitor Marimastat would reverse established steatosis, leading to improved outcome following hepatectomy.

**Methodology/Principal Findings:**

C57BL/6 male mice were fed a high fat diet for 9 weeks to establish obesity, hepatic steatosis and insulin resistance, and were administered either Marimastat or vehicle for an additional 2 or 4 weeks. Leptin deficient, hyperinsulinemic *ob/ob* mice were treated with Marimastat for 4 weeks. Hepatic steatosis was quantified by magnetic resonance spectroscopy and confirmed by histology. After two weeks, Marimastat-treated animals significantly improved surrogate markers for insulin sensitivity and liver histology, and experienced a 66% decrease in steatosis (*P* = 0.010). These findings were confirmed in *ob/ob* mice. Transcripts related to fatty acid synthesis were significantly downregulated in Marimastat-treated animals. Following pre-treatment with Marimastat or vehicle for two weeks, high fat fed C57BL/6 mice were subjected to two-thirds hepatectomy. Post-operative liver injury as quantified by serum aspartate aminotransferase levels and alanine aminotransferase levels was significantly decreased by 57% (*P* = 0.020) and 44% (*P* = 0.032) respectively, compared to controls.

**Conclusion/Significance:**

Treatment with the TACE-inhibitor Marimastat improved surrogate markers for insulin sensitivity and reversed steatosis in mouse models of diet-induced obesity and leptin deficiency, thereby attenuating post-operative injury following hepatectomy. This may suggest a potential therapeutic role in patients with fatty liver disease; especially those who need to undergo hepatic resection.

## Introduction

Non-alcoholic fatty liver disease (NAFLD) is one of the hallmarks of the metabolic syndrome and is a significant and increasingly more common cause of liver failure [1], [2]. NAFLD is characterized by an accumulation of triglycerides in hepatocytes (hepatic steatosis), and, together with lipotoxic injury, may lead to hepatocellular injury and inflammation (non-alcoholic steatohepatitis), advanced fibrosis, and ultimately liver failure [3]–[5]. Although its prevalence in both children and adults is increasing worldwide [1], [2], the mechanisms underlying the pathogenesis of NAFLD remain elusive.

Steatosis is an established risk factor for primary non-function of hepatic allografts [6] and for complications following major hepatic resection [7]. Patients with steatosis have up to a twofold increased risk of postoperative complications, and those with excessive steatosis (>30%) have an almost threefold increased risk of death [7]. Approximately 20% of patients undergoing liver resection and up to 25% of liver donors have some degree of steatosis, indicating the need for pharmacological intervention [8]. Currently, the cornerstone of clinical management is weight loss through diet and exercise [9]. In reality, however, surgical management usually does not allow time for such dramatic life style changes, justifying the search for a pharmacologic approach. Novel evidence suggests that vitamin E therapy and pioglitazone may be of use in patients with non-alcoholic steatohepatitis [10]. Pharmacological options to reverse steatosis prior to surgery, however, are lacking.

Insulin resistance and the resultant hyperinsulinemia, commonly associated with the metabolic syndrome, are involved in the imbalance of formation and utilization of free fatty acids leading to steatosis [11], [12]. In addition, visceral and hepatic lipid accumulation may activate inflammatory gene networks, suggesting a causal function of inflammation in the pathogenesis of the metabolic syndrome [13]. Tumor necrosis factor (TNF)-α is an inflammatory cytokine involved in linking nutrient availability to innate immune activation and is the major negative regulator of the insulin receptor pathway [14], [15] TNF-α release from the plasma membrane through its ectodomain shedding is mediated by TNF-α converting enzyme (TACE, also known as ADAM17 and CD156b), which belongs to the disintegrin and metalloproteinase family of zinc-metalloproteinases [16], [17] TACE is endogenously inhibited by tissue inhibitor of metalloproteinase (TIMP)-3 [18], and may be activated by metabolic stimuli such as hyperinsulinemia [19]. Mice deficient in TIMP-3 demonstrate elevated levels of TNF-α and develop insulin resistance and hepatic steatosis, mediated by increased TACE activity [20], [21]. In contrast, pharmacologic TACE-inhibition abrogates the inflammatory response and has been demonstrated to improve insulin resistance [22], [23].

The TACE/TIMP-3 system has recently emerged as a novel mediator between metabolic stimuli and innate immunity; however, the effects of pharmacologic TACE-inhibition on insulin resistance and hepatic steatosis remain to be established. We hypothesized that treatment with Marimastat would ameliorate insulin resistance and reverse established hepatic steatosis, potentially resulting in improved surgical outcome following hepatectomy in murine models of diet-induced obesity, hepatic steatosis and insulin resistance.

## Materials and Methods

### Ethics statement

Animal protocols complied with the National Institutes of Health Animal Research Advisory Committee guidelines and were approved by the Children's Hospital Boston Animal Care and Use Committee (protocol no. A06-08-065R).

### Animal experiments

Mice were housed 4 or 5 animals per cage on paper chip bedding in a barrier room with regulated temperature (21°C±1.6°C), humidity (45%±10%), and an alternating 12-hour light and dark cycle. The animals had free access to water and study diets, but a pair-feeding design was used after animals were assigned to the treatments to avoid bias resulting from a potential decreased caloric intake [24], [25].

**Figure 1:**
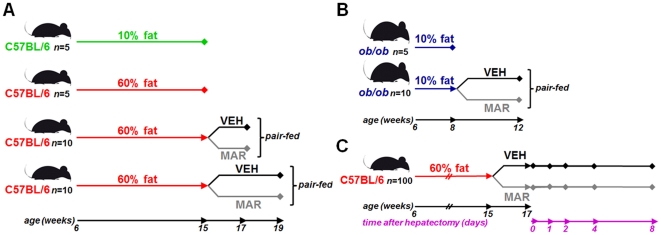
Design of the studies. Hepatic steatosis was induced by placing C57BL/6 mice on a 60% high fat diet for 9 weeks, followed by a two or four week treatment period with either Marimastat (MAR) or methylcellulose vehicle (VEH) twice daily (A). Leptin deficient ob/ob animals were placed on a 10% standard fat diet for two weeks, followed by a four week treatment period with either MAR or VEH twice daily (B). The efficacy of Marimastat on post-operative outcome following reversal of steatosis was evaluated in a model of two-thirds hepatectomy (C). C57BL/6 mice were placed on a 60% high fat diet, followed by two weeks of treatment with MAR or VEH twice daily. Mice were then subjected to a two-thirds hepatectomy, and sacrificed postoperatively to assess hepatic injury.

After an acclimation period of one week, twenty-five C57BL/6 male mice (#000664; Jackson Laboratories, Bar Harbor, ME) were placed on a high fat diet with 60% of calories derived from fat (#D12492; Research Diets, New Brunswick, NJ), whereas another group of five animals that were fed a standard purified rodent diet containing 10% of calories from fat (#D12450B; Research Diets) served as controls. Study diets were stored at −80°C and provided fresh each day to avoid lipid peroxidation. After 9 weeks, five animals from each group were sacrificed and tissues were analyzed to serve as baseline controls. The remaining twenty animals on the high fat diet were randomized to receive either 100 mg/kg of Marimastat (BB-2516, British Biotech, UK) in 0.45% methylcellulose (Sigma-Aldrich, St. Louis, MO) vehicle twice daily via orogastric gavage (MAR), or vehicle alone (VEH) for an additional two or four weeks (*n* = 5 per group). At the end of the treatment period, animals were sacrificed to evaluate hepatic steatosis and related parameters (**Figure 1A**).

In a separate experiment, fifteen B6.V-*Lep^ob^*/J male mice (#000632; Jackson Laboratories), commonly referred to as *ob/ob* mice [26], [27], were acclimatized for one week and placed on a standard purified rodent diet containing 10% of calories from fat. After two weeks, five animals were sacrificed to serve as base-line controls and the remaining animals were randomized to receive either Marimastat twice daily (MAR) or vehicle alone (VEH) for an additional four weeks (**Figure 1B**).

The efficacy of Marimastat to reverse steatosis and improve surgical outcome was studied using fifty C57BL/6 male mice placed on a similar high fat diet for 9 weeks, followed by two weeks of treatment with either Marimastat twice daily (MAR) or vehicle alone (VEH) while remaining on the high fat diet. After thirteen days, animals were withheld treatment for 24 hours to avoid potential inhibitory effects on liver regeneration [28]. The following morning, mice underwent the surgical removal of two-thirds of the liver under isoflurane anesthesia. The left upper, right upper, and left lower lobes of the liver were ligated with 5-0 silk ties (Ethicon, Somerville, NJ) and then excised, as previously described [29]. Animals were sacrificed either immediately to serve as controls, or after one, two, four and eight days to evaluate liver regeneration and related parameters (**Figure 1C**).

### Sample collection and serum biochemistry

At the end of the feeding experiments, mice were fasted for 6 hours. Glucose concentration was determined from tail vein blood using the OneTouch UltraSmart Blood Glucose Monitoring System (LifeScan, Milpitas, CA). Serum was delivered to the Clinical Laboratory at Children's Hospital Boston for analysis. The left lateral lobe was excised and collected for magnetic resonance (MR) spectroscopy analysis. White adipose tissue was dissected according to previously defined anatomic landmarks [24], [25]. A white adipose tissue fat-index was calculated using the sum of the individual fat pads as a percentage of the eviscerated body weight [24], [25].

### Surrogate index of insulin sensitivity and resistance

Insulin levels were measured using a rat/mouse insulin enzyme-linked immunosorbent assay kit (#EZRMI-13K; Millipore, St. Charles, MO). A surrogate index for insulin sensitivity was calculated using the log of homeostasis assessment (HOMA) and Quantitative insulin-sensitivity check index (QUICKI) formulas [24], [25], [30].

### Histology

Paraffin-embedded sections of the liver were stained by hematoxylin and eosin and periodic acid Schiff's/diastase to examine cellular architecture, glycogen deposition and lipid accumulation. Frozen tissue sections were stained with Oil Red-O to detect fat. A pathologist blinded to the treatment groups conducted a histological analysis of the liver sections.

### Magnetic resonance imaging

Magnetic resonance (MR) spectroscopy analysis was used to objectively quantify hepatic fat fraction as described previously [24], [25]. Briefly, samples were thawed at room temperature for 1 hour prior to analysis, blotted free of excess water and connective tissue, and placed in 5 mm diameter glass tubes for MR spectroscopy. An 8.5 T vertical bore magnet (DRX system, Bruker Instruments, Billerica, MA) was used for spectroscopic measurements of fat and water resonances. Specifically, a point resolved echo spectroscopic acquisition was applied to homogenous regions of liver, as identified from fast low angle shot images of the liver specimen. Voxel volumes interrogated spectroscopically with the point resolved echo spectroscopic sequence were 2 mm^3^. The repetition and echo times were 8 s and 12 ms, respectively, and 16 signal averages were acquired per spectrum. The water resonance and the methylene/methyl resonances were numerically integrated using the manufacturer supplied Paravision 4.0 software (Bruker Instruments). The methylene/methyl area was divided by the sum of the methylene/methyl area plus the water area to obtain the MR spectroscopy parameter representing hepatic fat fraction used for group comparisons.

### Cytokine and adipokine analysis

Serum levels of Adiponectin (#EZMADP-60K; Millipore), Leptin (#EZML-82K; Millipore), soluble TNF-α receptor II (p75) (#MRT20; Quantikine, R&D Systems, Minneapolis, MN) and IL-6 (#M6000B; Quantikine) were determined using commercial enzyme-linked immunosorbent assay kits. Optical density was read at 450 nm and analyzed with Softmax PRO Software (Molecular Devices, Sunnyvale, CA).

### Very low density lipoprotein secretion assay

After being fasted for 6 hours, mice were injected with 500 mg/kg tyloxapol (Triton-1339; Sigma-Aldrich) dissolved in saline at a concentration of 150 mg/mL via the tail vein. Both lipolysis and tissue uptake of lipoproteins are completely inhibited in mice under these conditions, and the accumulation of triglyceride in serum after injection of tyloxapol can be used to estimate rates of secretion of very low density lipoprotein [31]. Tail bleeds were performed just before injection (t_0_) and 45 and 90 min following injection. Serum was delivered to the Clinical Laboratory at Children's Hospital Boston for analysis of triglyceride levels. The slope of the line denotes the rate of VLDL production.

### TACE activity assay

Determination of TACE activity was performed using a commercially available α-secretase activity assay according to manufacturer's instructions (#FP001; R&D Systems, Minneapolis, MN). Briefly, 10 µL of recombinant human (#903-ADB; R&D Systems) and mouse (#2978-AD; R&D Systems) TACE were used as standards on a 96-well plate in triplicates, and increasing doses of Marimastat dissolved in H_2_O were added. After 1 hour, a TACE-specific peptide conjugated to the reporter molecules EDANS and DABCYL was added. In the uncleaved form, fluorescent emissions from EDANS were quenched by the physical proximity of the DABCYL moiety, which exhibits maximal absorption at the same wavelength (495 nm). Cleavage of the peptide by TACE physically separates the EDANS and DABCYL reporter molecules, allowing for the release of a fluorescent signal. The level of TACE enzymatic activity was proportional to the fluorometric reaction, measured at room temperature using the Wallac Victor2 Multilabel Counter (Perkin Elmer, Waltham, MA); with excitation at 355 nm, and emission at 510 nm.

### Analysis of mRNA expression

Isolation of mRNA, reverse transcription and quantitative real-time RT-PCR were performed as described previously [29], [34]. Relative transcript levels of genes of interest were quantified by real-time RT-PCR on a LightCycler 1.5 instrument (Roche, Mannheim, Germany) using the TaqMan methodology. TaqMan probes (dual-labeled with 5′-FAM and 3′-TAMRA) and primers were designed based on published sequences (**Table 1**) using the Primer Express software (Perkin Elmer), synthesized at MWG Biotech AG (Ebersberg, Germany), or acquired commercially (Life Technologies Corporation, Carlsbad, CA) [29], [34]. The housekeeping gene beta-2 microglobulin (β2MG) was amplified in parallel reactions for normalization.

**Table 1:** Primers and probes used in quantitative real-time RT-PCR.

Target gene	5′-Primer	TaqMan probe	3′-Primer
**Acc1**	TGTCCGCACTGACTGTAACCA	TCTTCCTCAACTTTGTGCCCACGGTC	TGCTCCGCACAGATTCTTCA
**Fasn**	CCTGGATAGCATTCCGAACCT	CCTGAGGGACCCTACCGCATAGC	AGCACATCTCGAAGGCTACACA
**Scd1**	CAACACCATGGCGTTCCA	TGACGTGTACGAATGGGCCCGA	GGTGGGCGCGGTGAT
**Elovl6**	GCGCTGTACGCTGCCTTTAT	TCGGCATCTGATGAACAAGCGAGC	GCGGCTTCCGAAGTTCAAA
**Srebp1**	CCAGAGGGTGAGCCTGACAA	CAATCAGGACCATGCCGACCTCT	AGCCTCTGCAATTTCCAGATCT
**Srebp2**	GCGGACAACACACAATATCATTG	AGCGCTACCGGTCCTCCATCA	TGACTAAGTCCTTCAACTCTATGATTTTG
**Ppara**	TGGTTCCTGGTGCCGATTTA	TGGTGGTAGATGCCTGCAACCCCA	ACTAGCATCCCACTTAATTATGTATCTGAA
**LXRa**	CGACAGAGCTTCGTCCACAA	CGGAAAAAGGGCCCAGCCCC	GCTCGTTCCCCAGCATTTT
**FXR**	CACGAAGATCAGATTGCTTTGC	CAAAGGGTCCGCAGTGGAGGCC	CCGCCGAACGAAGAAACA
**Mttp** *[Mm00435015_m1]*	*	*	*
**Apob** *[Mm01545156_m1]*	*	*	*
**Timp3** *[Mm00441827_m1]*	*	*	*
**TACE** *[Mm00456428_m1]*	*	*	*
**β2MG**	CTGATACATACGCCTGCAGAGTTAA	GACCGTCTACTGGGATCGAGACATGTG	ATGAATCTTCAGAGCATCATGAT

*Not given; commercially acquired from Life Technologies Corporation, Carlsbad, CA.

### Statistical analyses

Data are expressed as means ± standard error of the mean (SEM). Differences between two groups were assessed using the unpaired two-tailed Student's *t* test, applying Welch's correction when necessary. For non-parametric data the Mann-Whitney *U* test was used. Data sets involving more than two groups were assessed by analysis of variance. *P*<0.05 was considered statistically significant. All data were collected in a computerized Microsoft Excel database (Microsoft Inc., Redmond, WA). The analysis was performed with SPSS version 16.0 (SPSS Inc., Chicago, IL) statistical software, and figures were created using GraphPad Prism version 5.0 (GraphPad Software Inc., La Jolla, CA) software.

## Results

### Marimastat treatment reduced diet-induced hepatomegaly and normalized gross macroscopic appearance of livers from diet-induced obese mice

**Figure 2:**
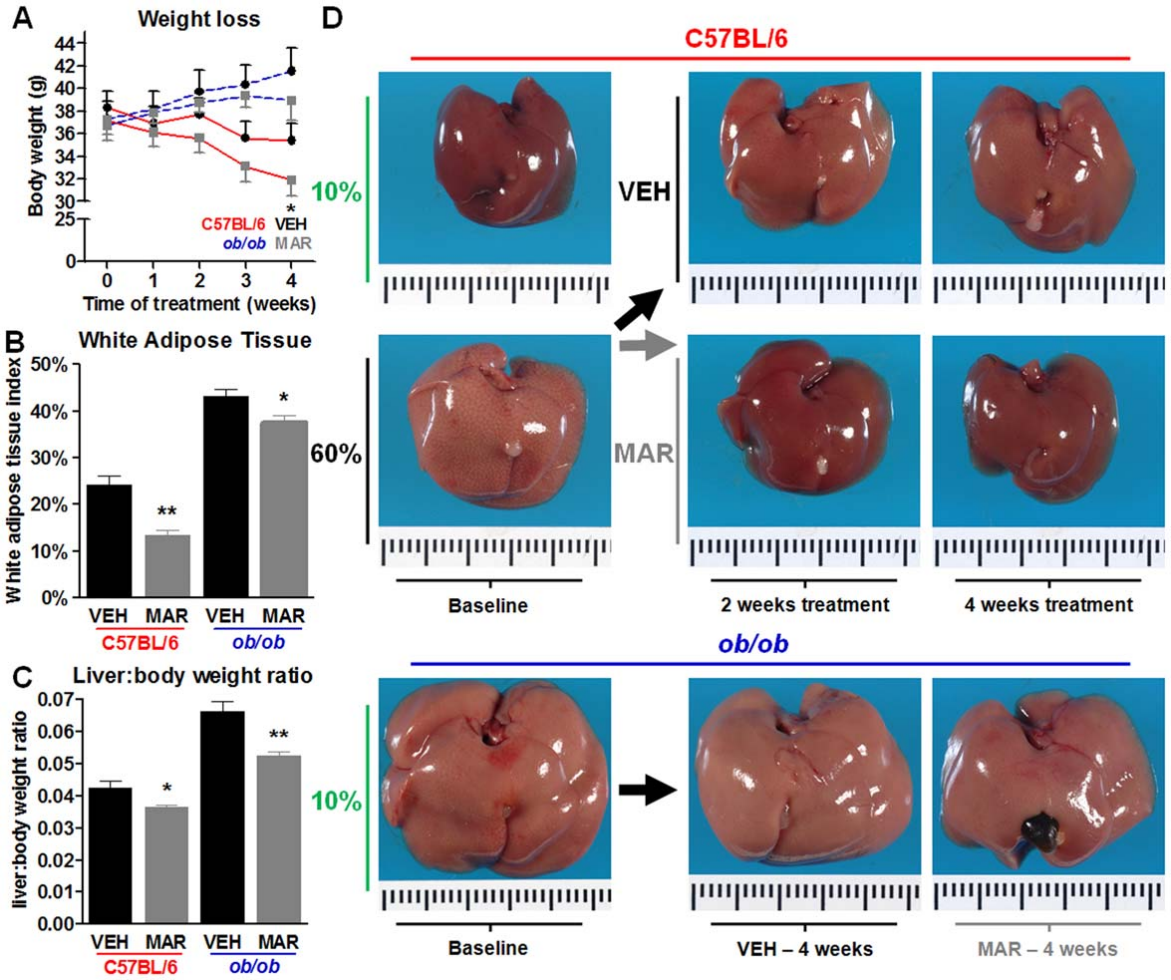
Marimastat treatment resulted in decreased adiposity and liver weights and normalized macroscopic appearance of the liver. Body weight loss was calculated relative to the weight of each individual animal before initiation of the experiment (A). As a measure of adiposity, white adipose tissue fat-index was calculated using the sum of the individual fat pads as a percentage of the eviscerated body weight (B). Liver to body weight ratios were calculated (C). Macroscopic appearance of livers from C57BL/6 and *ob/ob* mice at time of sacrifice (D). Values represent the mean ± SEM. Statistical significance is calculated between the VEH and MAR animals (*n* = 5 C57BL/6 and *n* = 4 *ob/ob* animals per group; *, *P*<0.05; **, *P*<0.01).

C57BL/6 animals fed a high fat diet for 9 weeks, followed by two or four weeks of treatment with Marimastat demonstrated subtle body weight loss, which was only significant after four weeks (**Figure 2A**). Body weights of *ob/ob* animals fed a normal fat diet followed by a four week course of Marimastat treatment remained unchanged throughout the study (**Figure 2A**). White adipose tissue fat index, as a measure of obesity, significantly decreased following Marimastat treatment in diet-induced obese mice as well as in *ob/ob* mice with 45% and 13%, respectively (*P* = 0.001 and *P* = 0.034; **Figure 2B**). Macroscopic appearance of livers from C57BL/6 mice fed a 10% fat purified rodent diet for 9 weeks demonstrated normal macroscopic liver appearance (**Figure 2B**; left upper pane). Baseline animals that were switched to a 60% fat diet for 9 weeks developed severe steatosis, indicated by liver enlargement and pale yellow discoloring (**Figure 2B**; left middle panel). Two and four weeks of Marimastat treatment resulted in significant improvement of macroscopic appearance (**Figure 2B**; middle panels), similar to the baseline control liver. Livers from vehicle treated mice remained pale yellow, indicative of fatty changes (**Figure 2B**; upper panels). Because of the marked differences already after two weeks, we present the data from this earlier time point throughout the rest of the manuscript. *Ob/ob* mice experienced severe liver enlargement and pale yellow discoloration, indicative for steatosis (left lower panel). Four weeks of Marimastat treatment resulted in minor improvement of macroscopic appearance compared to vehicle treated controls (lower panels). In both animal models, Marimastat treatment decreased liver to body weight ratios suggesting attenuation of hepatic steatosis (**Figure 2C**).

### Marimastat reversed hepatic steatosis and improved surrogate markers for insulin sensitivity in diet-induced obese mice as well as in leptin-deficient mice

**Figure 3:**
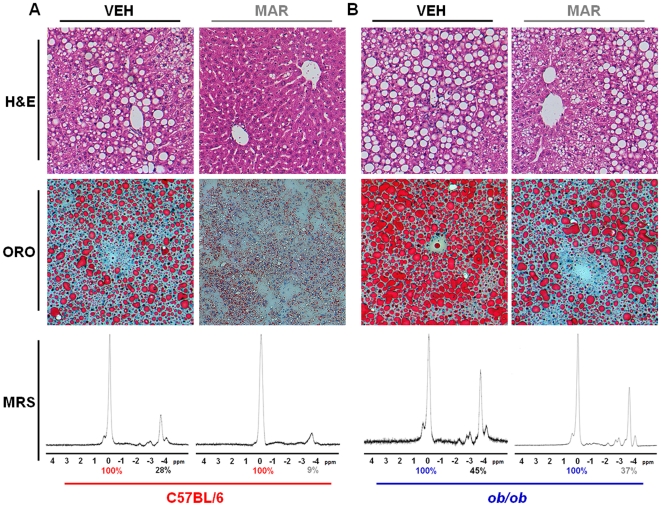
Marimastat reversed hepatic steatosis in diet-induced obese mice as well as in *ob/ob* animals, as demonstrated by histology and magnetic resonance spectroscopy. Representative liver sections stained with hematoxylin and eosin (H&E; top panels; original magnification 200×), and Oil Red O (ORO; middle panels; original magnification 200×). Hepatic fat fraction was measured by magnetic resonance spectroscopy (MRS; bottom panels, representative animals). Percent fat content was determined relative to water (100%) by numerical integration of the areas under the lipid and water peaks. Livers from MAR treated diet-induced obese animals exhibited almost normal hepatic architecture, whereas VEH livers revealed micro- and macrovesicular steatosis (A). Livers from VEH treated *ob/ob* mice revealed extensive, predominantly macrovesicular steatosis (B). MAR treated livers from *ob/ob* mice, in contrast, showed a decrease in macrovesicular lipid droplets mainly around the portal tract (B). Histological findings were confirmed with MRS, demonstrating a decrease in fat content in Marimastat-treated animals (A,B bottom panels).

**Figure 4:**
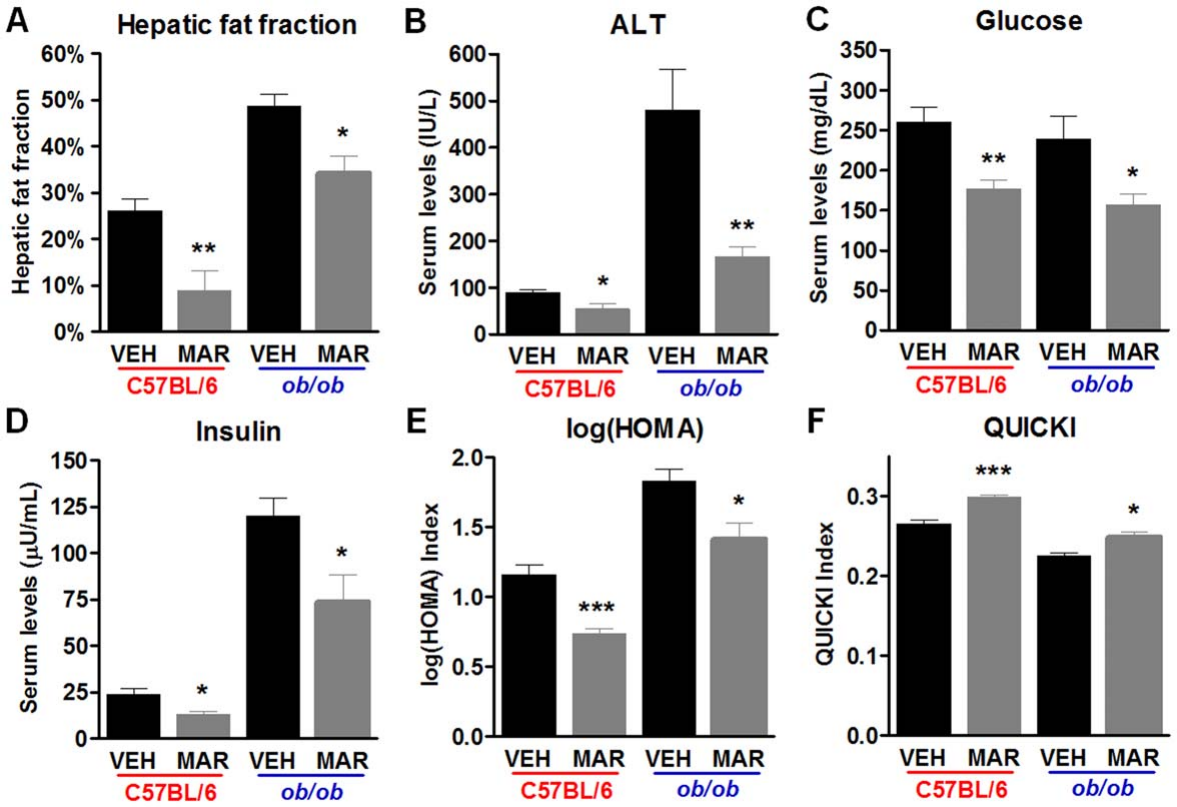
Marimastat reversed hepatic steatosis and improved surrogate markers for insulin sensitivity in diet-induced obese mice as well as in *ob/ob* animals. Mean hepatic fat fraction as measured by MR spectroscopy was decreased following Marimastat treatment (A). Marimastat treatment resulted in a decrease of plasma alanine aminotransferase levels (B), indicating decreased hepatic injury. Glucose (C) and insulin (D) levels in the fasted state, as well as log of homeostasis assessment (log(HOMA); E) and Quantitative insulin-sensitivity check index (QUICKI; F) as surrogate markers for insulin sensitivity were improved following treatment with Marimastat. Values represent the mean ± SEM. Statistical significance is calculated between the VEH and MAR animals (*n* = 5 C57BL/6 and *n* = 4 *ob/ob* animals per group; *, *P*<0.05; **, *P*<0.01; ***, *P*<0.001).

Liver sections from diet-induced obese animals treated for two weeks with Marimastat showed a significant reduction in steatosis, as demonstrated by H&E stains, and confirmed with Oil Red O staining (**Figure 3A**). Livers from Marimastat treated diet-induced obese mice exhibited almost normal hepatic architecture, whereas livers from control animals revealed micro- and macrovesicular steatosis. In *ob/ob* mice, Marimastat treatment led to a decrease in predominantly macrovesicular lipid droplets mainly around the portal tract, compared to livers from control animals that exhibited severe macrovesicular steatosis and ballooning (**Figure 3B**). To objectively confirm the histological findings, hepatic fat content was quantified by using MR spectroscopy (**Figure 3A,B** bottom panels). Two weeks of Marimastat treatment of diet-induced obese mice nearly normalized hepatic fat fraction to 9.0±4.3%, compared to control animals (26.1±2.6%, *P* = 0.010). In *ob/ob* animals, Marimastat treatment decreased hepatic fat fraction from 48.8±2.6% to 34.3±3.7% (*P* = 0.019) (**Figure 4A**). ALT was used as a marker for evaluation of hepatic injury associated with steatosis (**Figure 4B**). After two weeks of treatment with Marimastat, diet-induced obese animals exhibited a significant decrease of mean serum values for ALT (54±12 IU/L) than did control animals (89±7 IU/L; *P* = 0.035). Following four weeks of Marimastat treatment, ob/ob mice demonstrated a decreased mean ALT level of 166±20 IU/L, compared to control mice (480±87IU/L; *P* = 0.008). In addition, in the diet-induced obesity model as well as in *ob/ob* mice Marimastat treatment significantly lowered fasting glucose (*P* = 0.004 and *P* = 0.041, respectively; **Figure 4C**) and insulin (*P* = 0.013 and *P* = 0.039, respectively; **Figure 4D**) levels, resulting in improved surrogate markers for insulin sensitivity, as measured using the log(HOMA) (*P*<0.001 and *P* = 0.026, respectively; **Figure 4E**) and QUICKI (*P*<0.001 and *P* = 0.0307, respectively; **Figure 4F**) formulas. These data indicate that Marimastat treatment decreased liver injury and improved surrogate markers for insulin sensitivity in parallel with reversal of steatosis.

### Effects of Marimastat on serum adipokine levels and hepatic fatty acid export

**Figure 5:**
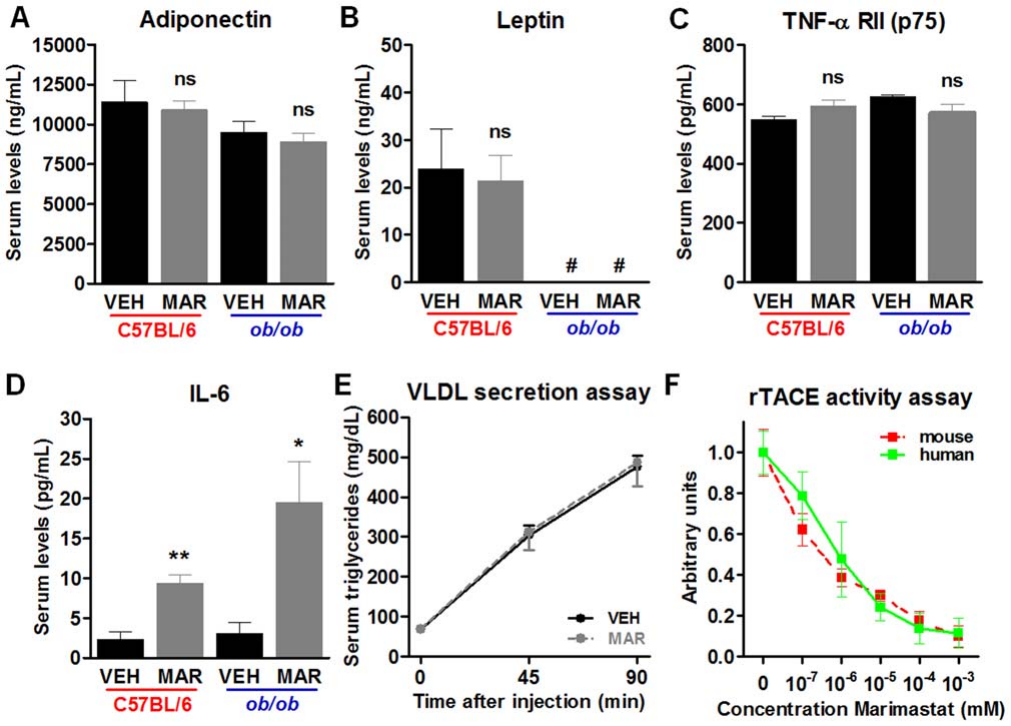
Effects of Marimastat on serum adipokine levels, fatty acid export from the liver and TACE activity. C57BL/6 animals were fed a high fat diet for 9 weeks, followed by two weeks of treatment with either Marimastat (MAR) or vehicle (VEH) control. *Ob/ob* animals were fed a normal fat diet and received 4 weeks of MAR or VEH control. MAR did not affect serum levels of adiponectin (A), leptin (B) and tumor necrosis factor-α receptor II (p75) (C); however, serum levels of IL-6 (D) were significantly increased in MAR animals in both studies. MAR did not affect hepatic lipid export rates, as quantified by injection of tyloxapol (E). MAR inhibited both human and mouse recombinant TACE in a dose-dependent matter. Values represent the mean ± SEM. Statistical significance is calculated between the VEH and MAR animals (*n* = 5 C57BL/6 and *n* = 4 *ob/ob* animals per group; *, *P*<0.05; **, *P*<0.01; ns, not significant).

To further explore the metabolic changes after Marimastat treatment, enzyme-linked immunosorbent assays were performed to detect serum levels of several adipokines. In both animal models, Marimastat treatment did not affect serum levels of adiponectin (**Figure 5A**), leptin (**Figure 5B**) or TNF-α receptor II (p75) (**Figure 5C**); however, serum levels of IL-6 (**Figure 5D**) were significantly increased. Because IL-6 is involved in hepatic export of triglyceride and cholesterol^32^, hepatic lipid export rates were quantified by injection of tyloxapol, an inhibitor of very low-density lipoprotein hydrolysis. Marimastat treatment did not affect serum triglyceride levels, indicating that Marimastat did not contribute to reversal of steatosis by increasing fatty acid export (**Figure 5E**).

### Marimastat treatment decreased TACE activity in vitro and decreased genetic feedback by Timp3

**Figure 6:**
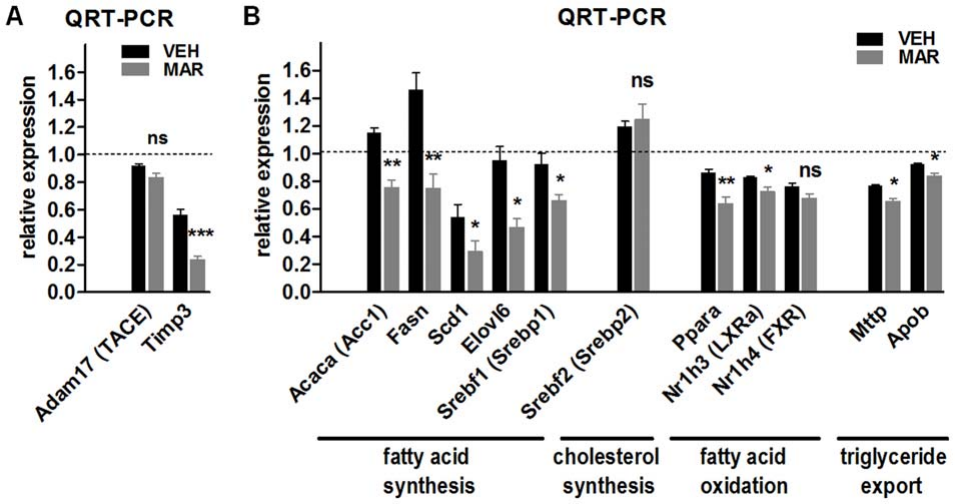
Relative mRNA expression of genes related to Timp3/TACE dyad (A) as well as expression of metabolic genes (B) in livers of mice fed a 60% high fat diet, comparing vehicle and Marimastat treatment at two weeks. Relative transcript levels were quantified by TaqMan quantitative RT-PCR, normalized by β2MG and expressed in arbitrary units *versus* 60% fat fed control animals at baseline (represented by the dotted line). Values represent the mean ± SEM. Statistical significance is calculated between the VEH and MAR animals (*n* = 5 animals per group; *, *P*<0.05; **, *P*<0.01; ***, *P*<0.001; ns, not significant).

To confirm inhibition of TACE by Marimastat, an activity assay was performed. This assay demonstrated efficient inhibition by Marimastat of both human and mouse recombinant TACE in a dose-dependent matter (**Figure 5F**). Quantitative real-time RT-PCR revealed that hepatic mRNA expression of TACE remained unchanged following Marimastat treatment; however, transcript levels of *Timp3*, the endogenous inhibitor of TACE, were decreased by 58% (**Figure 6A**). This suggests that Marimastat does not inhibit TACE at the gene level, but that inhibition of TACE activity seizes the need for endogenous inhibition, resulting in decreased mRNA expression of *Timp3*.

### Hepatic mRNA expression analysis of metabolic genes, as quantified by real-time RT-PCR

To investigate the changes in metabolic gene signaling induced by Marimastat treatment, hepatic transcript levels of metabolic genes were examined using real-time RT-PCR (**Figure 6B**). Marimastat treatment significantly downregulated genes involved in fatty acid synthesis such as acetyl-coenzyme a carboxylase alpha (*Acaca*), fatty acid synthase (*Fasn*), stearoyl-coenzyme a desaturase 1 (*Scd1*), elongation of long chain fatty acids family member 6 (*Elovl6*) and sterol regulatory element binding factor 1 (*Srefp1*), whereas levels of *Srebf2*, key transcription factors involved in cholesterol synthesis, remained unaffected. Marimastat treatment slightly decreased hepatic transcript levels of genes related to fatty acid oxidation such as peroxisome proliferator activated receptor alpha (*Ppara*), liver X receptor alpha (*Nr1h3*) and farnesoid X receptor (*Nr1h4*), as well as genes involved in triglyceride export such as microsomal triglyceride transfer protein (*Mttp*) and apolipoprotein b (*Apob*). These findings indicate that the improvement in insulin resistance, steatosis and inflammation by Marimastat treatment is associated with inhibition of genes related to fatty acid synthesis, rather than genes involved in cholesterol synthesis, fatty acid oxidation or fatty acid export.

### Short-term reversal of established steatosis using Marimastat prior to hepatic resection ameliorated post-operative liver injury, without affecting regeneration

**Figure 7:**
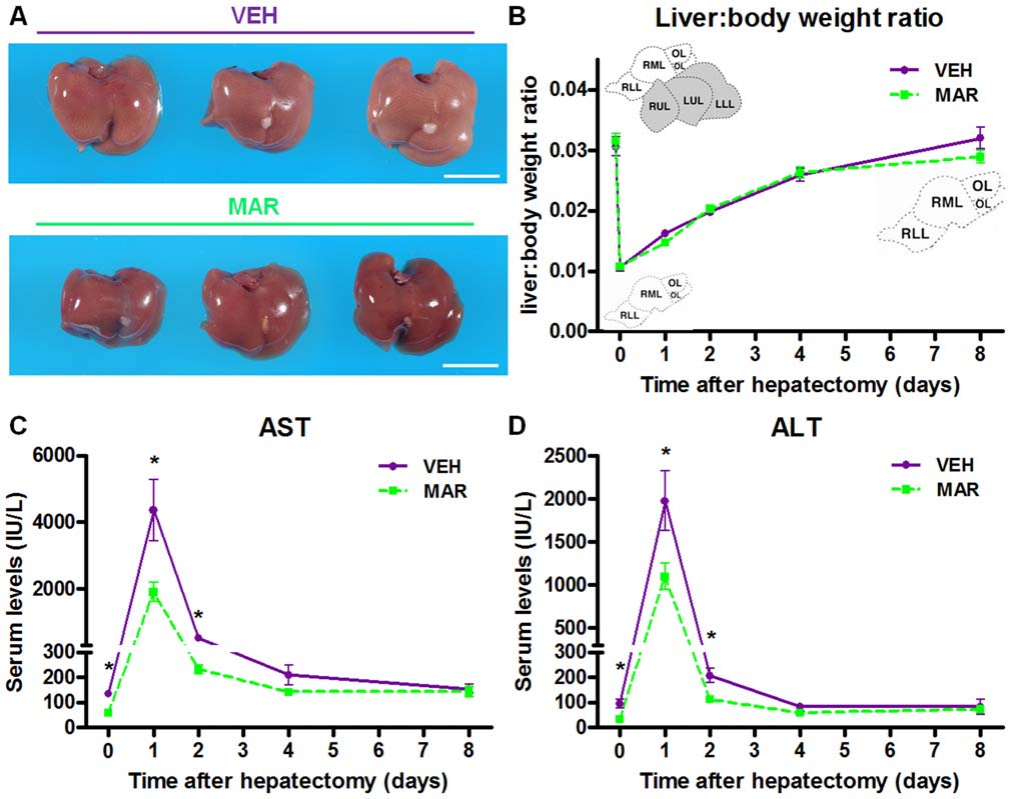
Reversal of established steatosis using Marimastat ameliorated post-operative liver injury, without affecting regeneration. C57BL/6 animals were fed a high fat diet for 9 weeks, followed by two weeks of treatment with either Marimastat (MAR) or vehicle (VEH) control. After reversal of steatosis (A), animals were subjected to a two-thirds hepatectomy. Marimastat did not affect speed of liver regeneration, as measured by liver to body weight ratios over time (B). Post-operative liver injury was significantly decreased in diet-induced obese animals that had their steatosis reversed following Marimastat treatment, as quantified by aspartate aminotransferase (AST; C) and alanine aminotransferase (ALT; D). Values represent the mean ± SEM. Statistical significance is calculated between the VEH and MAR animals (*n* = 10 animals per group per time point; *, *P*<0.05; **, *P*<0.01; ***, *P*<0.001).

To further explore the potential clinical benefits of Marimastat, we studied whether pre-treatment with a short course of Marimastat to reverse hepatic steatosis and insulin resistance prior to surgery (as described above) would improve the outcome of liver resection in the context of fatty liver. To address this question, we utilized a model of two-thirds hepatectomy, which was performed in diet-induced obese animals with hepatic steatosis, pre-treated with Marimastat or not as described above. Indices of liver regeneration and injury were evaluated as surrogate predictors of post-operative outcomes. As expected, two weeks of treatment with Marimastat successfully reversed steatosis, whereas livers of vehicle-treated animals remained fatty (**Figure 7A**). Importantly, speed of liver regeneration was similar in both groups, as determined by the restoration of liver to body weight ratios in the eight days following surgery (**Figure 7B**). More strikingly, post-operative liver injury as quantified by AST (**Figure 7C**) and ALT (**Figure 7D**) was significantly decreased by 57% (*P* = 0.020) and 44% (*P* = 0.032), respectively, after 24 hours, and by 55% (*P* = 0.036) and 45% (*P* = 0.022), respectively, after 48 hours in the group of diet-induced obese animals that had their steatosis reversed following Marimastat treatment, compared to controls. In both groups, AST and ALT levels normalized after four to eight days. Post-operative mortality was limited to one death in each group related to technical failure. These data indicate that the reversal of steatosis with Marimastat significantly attenuated post-operative injury following hepatectomy.

## Discussion

The present study aimed to determine the effects of the broad-spectrum matrix metalloproteinase and TACE-inhibitor Marimastat on hepatic steatosis and insulin resistance in murine models of diet-induced obesity and leptin deficiency. In addition, we studied the surgical outcome of treated versus untreated animals following hepatectomy. We demonstrate that Marimastat treatment improves surrogate markers for insulin sensitivity and reverses steatosis in both animal models by interfering, in all likelihood, with TACE activation and signaling. The beneficial effects of steatosis reversal on post-operative outcome by pharmacologic TACE-inhibition are encouraging, and provide a rationale for current investigations in the search for a pharmacologic agent to optimize the steatotic liver prior to surgery. For this indication, a drug such as Marimastat with its known safety profiles and limited side-effects would allow rapid transfer to clinical practice.

Previous reports have implicated the TACE/TIMP-3 dyad as a mediator between metabolic stimuli and innate immunity. Animals double-heterozygous for the *insulin receptor* and *Timp3* developed spontaneous mild hyperglycemia and hyperinsulinemia at three months, and overt glucose intolerance and hyperinsulinemia at six months [33]. Mice heterozygous for *Tace* are protected from diet-induced insulin resistance and diabetes [34], whereas pharmacologic TACE-inhibition reduced hyperglycemia and vascular inflammation in diabetic mice heterozygous for the insulin receptor [33], as well as in fructose-fed rats [23]. In contrast, *Timp3* deficient animals fed a high fat diet for five months experienced increased TACE activity, became glucose-intolerant and insulin-resistant and developed severe macrovesicular steatosis compared to wild-type mice [20]. In another study, mice double-heterozygous for the *insulin receptor* and *Timp3* that were placed on a high fat diet developed macrovesicular steatosis and showed a higher degree of hepatic and adipose tissue inflammation compared to wild-type mice [21]. In contrast, mice double-heterozygous for the *insulin receptor* and *Tace* had a nearly opposite phenotype [21]. These data indicate that the interplay between reduced insulin action and increased TACE activity promotes diabetes, vascular inflammation and hepatic steatosis. Until now, the effects of pharmacologic TACE-inhibition on hepatic steatosis remained to be established. Therefore, we aimed to investigate the effects of TACE-inhibition in two commonly used models of obesity and hepatic steatosis. In the diet-induced obesity model, we show that two weeks of Marimastat treatment almost completely reverses the metabolic phenotype, compared to vehicle-treated obese controls. These findings were validated in a second, mechanistically different animal model of obesity and hepatic steatosis using the murine model of leptin deficiency.

Adipokines, a group of polypeptide molecules primarily secreted by adipose tissue exert local, peripheral and/or central actions and are involved in the pathogenesis of NAFLD [35]. The cytokines TNF-α and IL-6 are involved in induction of insulin and leptin resistance and can be stimulated by the adipokine leptin (regulated by the *ob* gene), or inhibited by the adipokine adiponectin [35]. TACE, in part, regulates the production of TNF-α and IL-6, and may indirectly affect release of adiponectin and leptin. Our data demonstrates that despite efficient inhibition of TACE activity by Marimastat, serum levels of the adipokines, adiponectin, leptin and TNF-α receptor II remained unchanged. In contrast, serum levels of IL-6 were significantly elevated following Marimastat treatment. Because IL-6 is also involved in hepatic triglyceride secretion [32], we hypothesized that the increase in IL-6 could have contributed to the improvement of hepatic steatosis by regulating hepatic fatty acid export by the liver. To address this, we quantified hepatic lipid export by injecting tyloxapol, an inhibitor of very low-density lipoprotein hydrolysis, and measured serum triglyceride levels. The rate of very low-density lipoprotein production was similar in both groups, indicating that Marimastat treatment did not affect hepatic fatty acid export.

Pathways and mechanisms linking the TACE/TIMP-3 dyad to insulin resistance and hepatic steatosis may involve expression of proteins playing a role in fatty acid uptake, triglyceride synthesis and methionine metabolism [20]. Hepatic gene expression analysis in our study indicates that Marimastat treatment significantly decreased transcript levels of genes related to fatty acid synthesis, such as *Acaca*, *Fasn*, *Scd1*, *Elovl6* and *Srebf1*, while *Srepf2*, a gene involved in cholesterol synthesis, remained unchanged. Hepatic transcript levels of genes involved in fatty acid oxidation such as *Ppara*, *Nr1h3* and *Nr1h4* were slightly, but significantly, decreased following Marimastat treatment. This was similar for hepatic gene expression of *Mttp* and *Apob*, both involved in triglyceride export. This data suggests that a significant inhibition of hepatic fatty acid synthesis, in combination with relatively unchanged rates of fatty acid oxidation and triglyceride export, may be, at least in part, responsible for the beneficial effects of Marimastat in the reversal of hepatic steatosis.

In a previous study, mice deficient in *Timp3* had increased activity of TACE and elevated levels of TNF, and developed spontaneous, severe inflammation in the liver [36]. In addition, following partial hepatectomy these mice succumbed to liver failure, indicating the importance of TNF signaling in liver regeneration [36]. In line with this data, we have previously demonstrated that matrix metalloproteinases and TACE-inhibition by Marimastat significantly attenuated hepatic injury and inflammation following carbon tetrachloride intoxication [37], but may impair hepatic regeneration when administered concomitantly [28]. Also, in a murine model of fat free diet-induced essential fatty acid deficiency, we have demonstrated that Marimastat treatment markedly prevented hepatic triglyceride accumulation, despite elevated rates of *de novo* lipogenesis and onset of essential fatty acid deficiency [38]. In the current paper, Marimastat treatment ameliorated hepatic injury in both diet-induced obese mice as well as in leptin deficient *ob/ob* animals, corroborating our previous findings. More importantly, in the first post-operative days following two-thirds hepatectomy in diet-induced obese mice, Marimastat pre-treatment reversed hepatic steatosis and decreased hepatic injury by about 50%. Liver growth rate was not impaired, because Marimastat treatment was halted 24 hours prior to hepatic resection to avoid any potential effects of TACE-inhibition on hepatic regeneration [28]. These findings may deserve further experimental investigation in the search for a pharmacologic treatment for the reversal of steatosis.

Although we cannot extrapolate our experimental *in vivo* results with Marimastat to human clinical trials directly, it is possible that Marimastat as well as other drugs with anti-TACE activity may have similar effects on hepatic steatosis. Clinically, Marimastat has been used in multiple oncologic clinical trials up to phase III; however, its overall therapeutic benefit was limited and the trials were halted [39]–[41]. A final note of caution is warranted when patients with chronic, active liver diseases such as hepatitis or non-alcoholic steatohepatitis receive long-term treatment with TACE and matrix metalloproteinase-inhibitors, since inhibition of fibrolytic matrix metalloproteinases will likely accelerate hepatic fibrogenesis formation [37]. However, this is unlikely to cause a problem in a short term treatment mode prior to surgery. Nonetheless, the use of these drugs could still be used in those with simple steatosis or for short periods of time, since the effects on fibrogenesis may only apply to long-term use [37].

In conclusion, we demonstrated that TACE-activity inhibition by Marimastat in two murine models of hepatic steatosis resulted in reversal of steatosis, coupled with improvement of surrogate markers of insulin sensitivity. Short-term use of Marimastat decreased hepatic fat content of high fat-fed obese mice, and subsequently attenuated hepatic injury following major hepatic resection. Since effective inhibition of TACE activity improved surgical outcome, our data suggests a potential use in patients with steatosis who need to undergo major hepatic resection. Based on these findings in animal models, we advocate further investigation of pharmacologic TACE-inhibitors in human patients affected by NAFLD.
